# Role of platelet activation in the cardiovascular complications associated with HIV infection: differential effect of abacavir versus tenofovir

**DOI:** 10.1186/1758-2652-13-S4-P62

**Published:** 2010-11-08

**Authors:** D Francisci, E Falcinelli, B Belfiori, E Petito, M Mezzasoma, F Baldelli, P Gresele

**Affiliations:** 1 University of Perugia, Division of Infectious Diseases, Department of Ex, Perugia, Italy; 2 University of Perugia, Division of Internal and Cardiovascular Medicine,, Perugia, Italy

## Purpose

Abacavir use has been associated with an increased risk of ischemic cardiovascular events in several cohort studies, but the pathogenic mechanisms are still unknown. Recently Martinez et al. observed no differences in markers of inflammation, endothelial dysfunction, insulin resistance or hypercoagulability in HIV infected persons treated with either abacavir or tenofovir. While several studies have shown endothelial dysfunction in HIV-infected patients, only a few data are available on the involvement of platelets. Aim of our study was to evaluate markers of platelet activation and endothelial dysfunction in HIV infection comparing patients treated with abacavir or tenofovir.

## Methods

In a retrospective, case-control study, the time course of some endothelial (MCP-1, sVCAM-1) and platelet activation markers (sPLA2, sGPV, sP-sel) was examined in 62 HIV-infected patients, before starting HAART and after 6-12 months of therapy with either Abacavir (n=31) or Tenofovir (n=31). Data were compared with those from 20 untreated HIV-infected patients at diagnosis and after 6 months and 10 healthy matched controls.

## Results

Soluble P-selectin (sP-sel), sPLA2, soluble vascular cell adhesion molecule-1 (sVCAM-1) and monocyte chemoattractant protein-1 (MCP-1) were significantly higher in HIV-infected patients than in healthy controls. During 6-12 months of follow-up, we found no significant differences between abacavir and tenofovir-treatment for endothelial markers, while sPLA2 (2113.6±39.5 vs 2742.5±29.7pg/ml, p<0.05) and sPsel (524±53.2 vs 713.47±20.3ng/ml, p<0.05) increased significantly in the abacavir group as compared with the tenofovir-treated group. sGPV, the platelet glycoprotein V major fragment released upon thrombin cleavage, was increased after 6-12 months as compared to baseline in both treatment groups (Abacavir: 89.7±13.7 vs 118.64±15.3 ng/ml Tenofovir: 133.5±10.7 vs 157.36±11.2, p<0.05). In naïve patients, not treated with HAART, significantly increased plasma markers of endothelial dysfunction sPsel and sPLA2 were confirmed at diagnosis, as compared with healthy controls, with no changes upon follow-up.

## Conclusions

Our results confirm that chronic HIV infection induces endothelial dysfunction but indicate that a short-term treatment with abacavir enhances also some parameters of platelet activation, suggesting a role of platelets too in the increased incidence of ischemic cardiovascular events in HIV-infected patients treated with abacavir*.*

